# Supplementary material for: E3 ubiquitin ligase RNF10 promotes dissociation of stalled ribosomes and responds to ribosomal subunit imbalance
Source: Nat Commun. 2024 Nov 28;15:10350. doi: 10.1038/s41467-024-54411-x (PMC11604940; doi:10.1038/s41467-024-54411-x)
Supplement: Supplementary file 1 — Supplementary Information [file 41467_2024_54411_MOESM1_ESM.pdf]

Supplementary Information

E3 ubiquitin ligase RNF10 promotes dissociation of stalled ribosomes and responds to ribosomal subunit imbalance

Janina A. Lehmann[1,2], Doris Lindner[1,2], Hsu-Min Sung[1,2], Georg Stoecklin[1, 2]

[1] Division of Biochemistry, Mannheim Institute for Innate Immunoscience (MI3) and Mannheim Cancer Center (MCC), Medical Faculty Mannheim, Heidelberg University, 68167 Mannheim, Germany

[2] Center for Molecular Biology of Heidelberg University (ZMBH), German Cancer Research Center (DKFZ)-ZMBH Alliance, 69120 Heidelberg, Germany

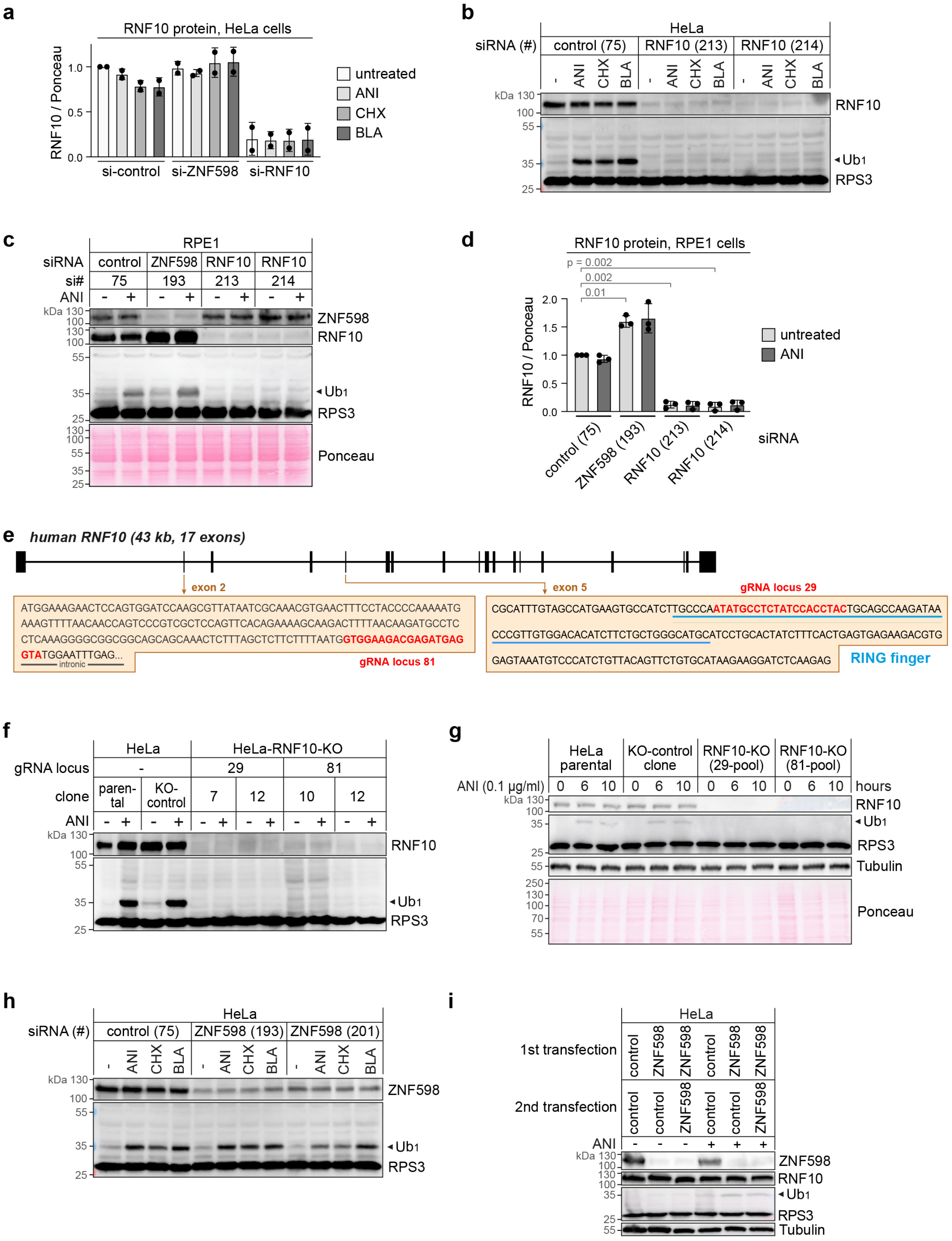

Supplementary Fig. 1 | RNF10 mediates RPS3 monoubiquitination.

Legend to Supplementary Fig. 1.

a RNF10 protein levels were quantified from Western blots as in (**Fig. 1a**), shown are mean values ± SD (n = 2). **b** Following control KD or KD of RNF10 with two different siRNAs for 72 h, HeLa cells were treated for 2 h with translation elongation inhibitors ANI (0.1 µg/ml), CHX (100 µg/ml) or BLA (100 µg/ml). Ub_1-RPS3 was monitored by Western blot analysis. The blot is representative of 2 independent experiments. **c** Western Blot analysis of Ub_1-RPS3 in RPE1 cells 72 h after control KD, KD of ZNF598 or KD of RNF10; ± ANI treatment (2 h, 0.1 µg/ml). The blot is representative of 3 independent experiments. **d** RNF10 protein levels were quantified from Western blots as in panel (**c**), shown are mean values ± SD (n = 3 biological replicates; p values determined by two-tailed, paired t test). **e** Schematic illustration of the CRISPR/Cas9 strategy for RNF10 KO showing gRNA locus 81 on exon 2 and locus 29 on exon 5 of the human *RNF10* gene. **f** Western blot analysis showing the effect of ANI treatment (2 h, 0.1 µg/ml) on Ub_1-RPS3 in RNF10-KO HeLa cells (clones 29-7, 29-12, 81-10, 81-12) compared to parental HeLa cells and a non-targeting KO control clone. The blot is representative of ≥ 2 independent experiments. **g** Western blot analysis of RNF10 and Ub_1-RPS3 in HeLa-RNF10-KO pools compared to parental HeLa and KO-control cells 0, 6, or 10 hours after ANI treatment (0.1 µg/ml). **h** Following control KD or KD of ZNF598 with two different siRNAs for 72 h, HeLa cells were treated for 2 h with translation elongation inhibitors ANI (0.1 µg/ml), CHX (100 µg/ml) or BLA (100 µg/ml). Ub_1-RPS3 was monitored by Western blot analysis. The blot is representative of two independent experiments. **i** Western blot analysis of ZNF598 and RNF10 after KD of ZNF598 (S193, 24 h) followed by a second KD of ZNF598 (24 h) ± ANI treatment (2 h before harvest, 0.1 µg/ml). The blot is representative of 2 independent experiments. In panels (**c**, **g**, **i**), Tubulin or Ponceau staining serve as loading controls. Source data are provided as a Source Data file.

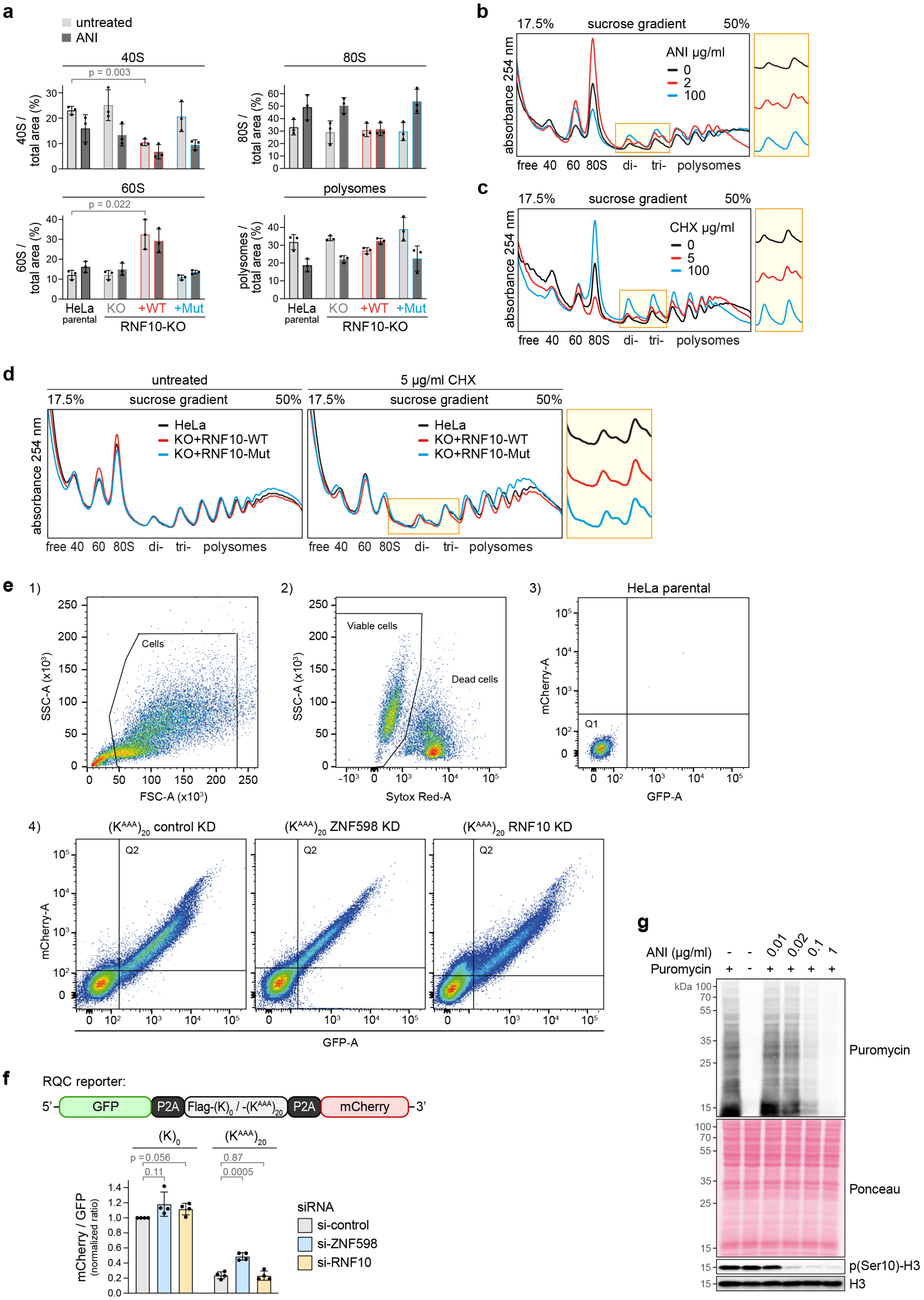

Supplementary Fig. 2 | Ribosomal half-mer formation and RQC analysis.

Legend to Supplementary Fig. 2.
a Polysome profiles as in (**Fig. 2b**) were quantified by calculating the area under the 40S, 60S, and 80S peaks as well as under the polysomal part of the profile, shown are mean values ± SD (n = 3 biological replicates; p values determined by two-tailed, paired t test). **b** Polysome profile analysis of HeLa cells treated with different ANI concentrations for 2 h. **c** Polysome profile analysis of HeLa cells treated with different CHX concentrations for 2 h. **d** Polysome profile analysis of HeLa-RNF10-KO cells (clone 29-7) stably expressing HA-RNF10-WT (KO+RNF10-WT) or HA-RNF10-Mut (C225S/C228S) (KO+RNF10-Mut), ± CHX treatment (2 h); the yellow area is enlarged on the right side. **e** FACS analysis: 1) FSC-A/SSC-A gating of HeLa single cell population, excluding debris and doublets; 2) Sytox Red APC-A/SSC-A gating into viable and dead cells; 3) GFP-A/mCherry-A gating of viable non-transfected HeLa control cells in quadrant Q1; 4) GFP-A/mCherry-A gating of viable HeLa cells transfected with siRNA (si-control (S75), si-ZNF598 (S193), si-RNF10 (S213)) and the $(K^{AAA})_{20}$ RQC reporter gene into GFP and mCherry double positive cells in quadrant Q2, excluding autofluorescent GFP and mCherry double negative cells. **f** Schematic illustration showing the RQC reporter genes that separately expresses GFP, FLAG-$(K)_0$ or FLAG-$(K^{AAA})_{20}$, and mCherry, through skipping of peptide bond formation at the P2A sequence. Following KD of ZNF598 (siRNA S193) or RNF10 (S213), or control KD (S75) for 14 h, HeLa cells were transfected with the RQC reporter genes. After 48 h, cells were subjected to FACS analysis for quantification of the mCherry / GFP ratio, shown are mean values ± SD (n = 4 biological replicates; p values determined by two-tailed, paired t test). **g** HeLa cells were treated with DMSO (negative control) or ANI at the indicated concentrations for 2 h. Puromycin (10 ug/ml) was added 10 minutes before cell lysis, and Western blot analysis was carried out using antibodies against puromycin, phospho(serine 10)-histone H3 and total histone H3. The blot is representative of 3 independent experiments. Signal quantification is shown in (**Fig. 2f**). Source data are provided as a Source Data file.

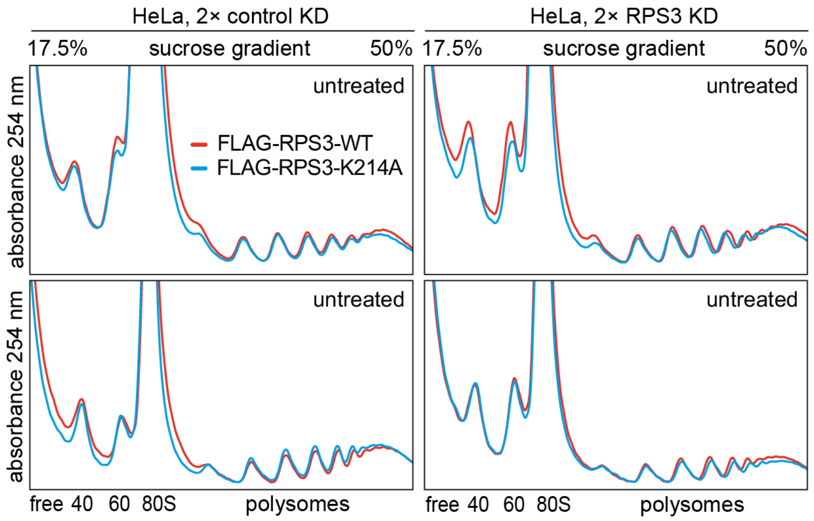

Supplementary Fig. 3 | Control polysome profiles.
Controls to (**Fig. 3b**) showing polysome profiles of HeLa cells stably expressing FLAG-RPS3-WT or FLAG-RPS3-K214A after KD of RPS3 (siRNA S292) or control KD (S75) on day 1 followed by a second KD of RPS3 or control KD on day 3. Polysome profiles were recorded on day 5 (upper panels, controls to 0.1 µg/ml ANI treatment) or on day 6 (lower panels, controls to 0.02 µg/ml ANI treatment).

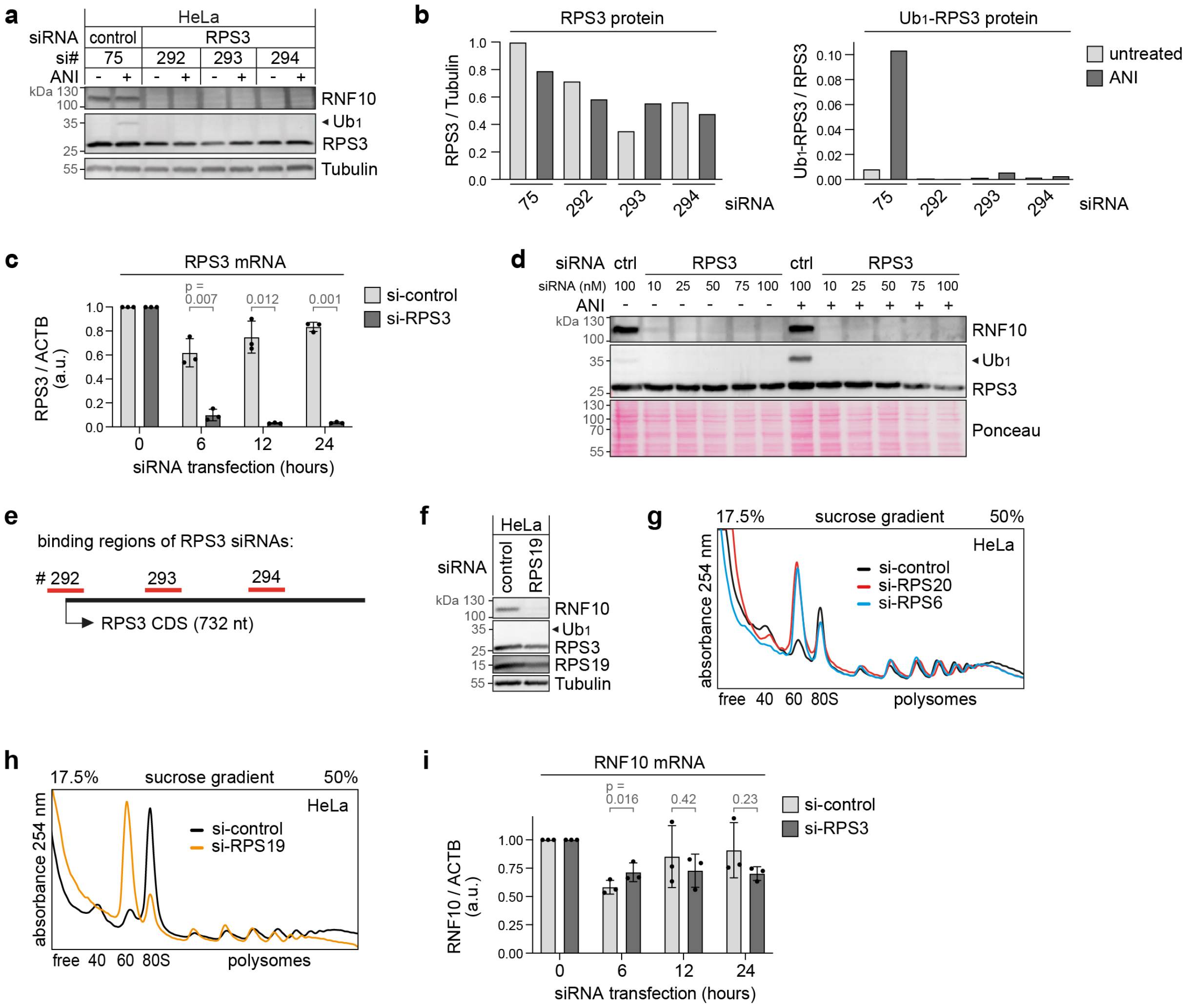

Supplementary Fig. 4 | Impaired 40S ribosome biogenesis leads to RNF10 degradation.
a Western blot analysis of RNF10 and Ub_1-RPS3 in HeLa cells 24 h after KD of RPS3 using three different siRNAs in comparision to control KD, ± ANI treatment (2 h, 0.1 µg/ml). Tubulin serves as loading control. **b** RPS3 and Ub_1-RPS3 protein levels were quantified from the Western blot in panel (**a**). **c** RPS3 mRNA levels were determined by RT-qPCR analysis in HeLa cells following KD of RPS3 (siRNA S292) for different periods of time. Values were normalized to β-actin (ACTB) mRNA (mean ± SD; n = 3 biological replicates; p values determined by two-sided, paired t test). **d** Western blot analysis of RNF10 and Ub_1-RPS3 in HeLa cells 24 h after KD of RPS3 (S292) with different siRNA concentrations, ± ANI treatment (0.1 µg/ml, 2 h); Ponceau staining serves as loading control. **e** Scheme of the RPS3 coding sequence (CDS) showing the binding regions of three different siRNAs against RPS3. **f** Western blot analysis of RNF10 and Ub_1-RPS3 in HeLa cells 24 h after control KD (S75) or KD of RPS19 (S309). Tubulin serves as loading control. The blot is representative of 3 independent experiments. **g** Polysome profile analysis of HeLa cells 12 h after KD of RPS20 (S297) or RPS6 (S299) in comparison to control KD (S75). **h** Polysome profile analysis of HeLa cells 12 h after KD of RPS19 (S309) in comparison to control KD (S75). **i** RNF10 mRNA levels were determined by RT-qPCR analysis in HeLa cells following KD of RPS3 (S292) for different periods of time. Values were normalized to ACTB mRNA (mean ± SD; n = 3 biological replicates; p values determined by two-sided, paired t test). Source data are provided as a Source Data file.

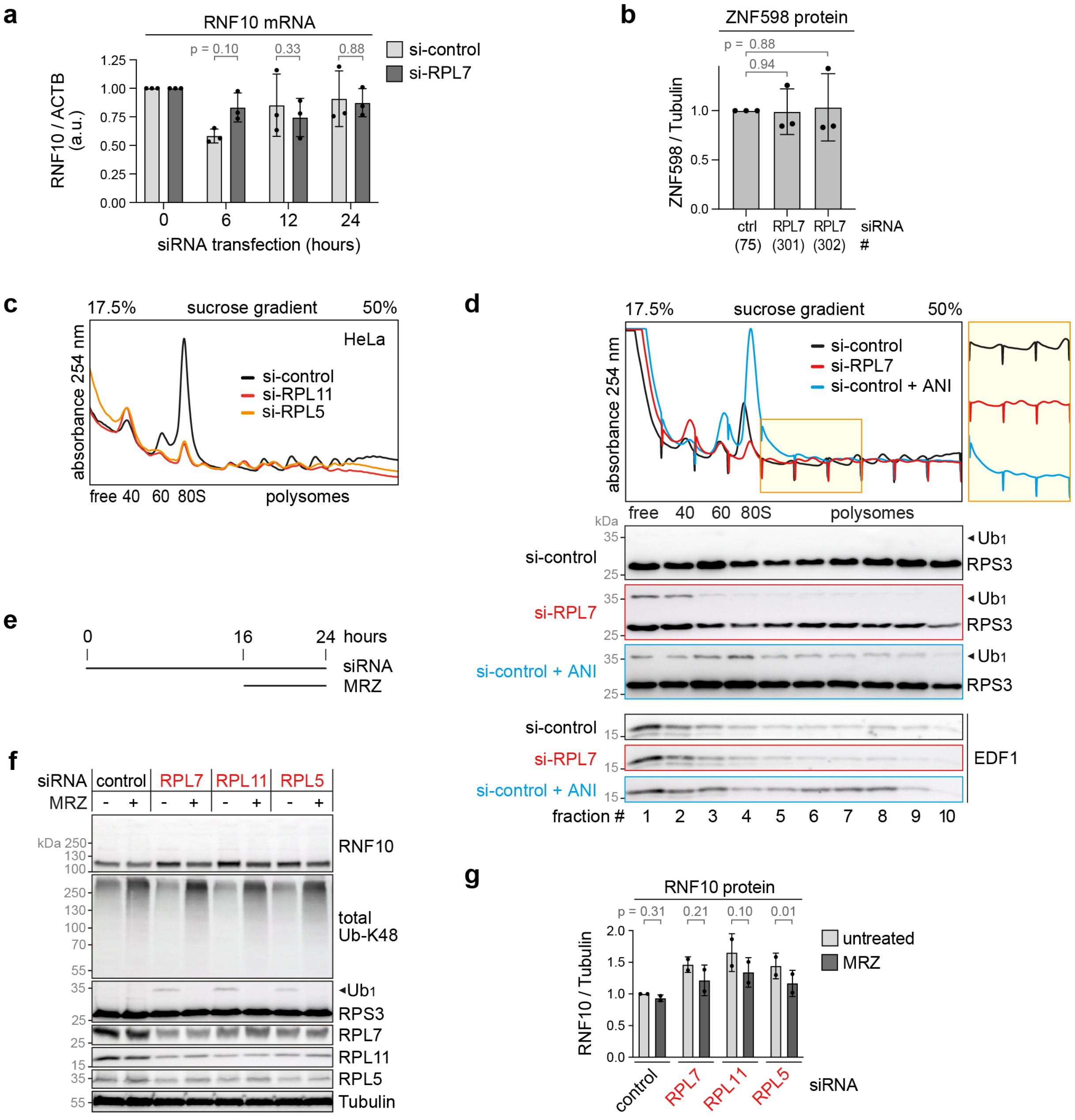

Supplementary Fig. 5 | Impairment of 60S biogenesis by RPL KD.
a RNF10 mRNA levels were determined by RT-qPCR analysis in HeLa cells following KD of RPL7 (siRNA S301) for different periods of time. Values were normalized to ACTB mRNA (mean ± SD; n = 3 biological replicates; p values determined by two-sided, paired t test). **b** ZNF598 protein levels were quantified from Western blots as in (**Fig. 5b**), shown are mean values ± SD (n = 3 biological replicates; p values determined by two-tailed, paired t test). **c** Polysome profile analysis of HeLa cells 12 h after KD of RPL11 (S304) or RPL5 (S310) in comparison to control KD (S75). **d** Polysome profile and fractionation analysis of HeLa cells 24 h after KD of RPL7 (S301) in comparison to control KD (S75) ± ANI treatment (2 h, 0.1 µg/ml). Sharp peaks pointing downwards are artefacts of the electric signal of the fractionator. Profiles are shown separately in the yellow area on the right side. The distribution of Ub$_1$-RPS3, RPS3 and EDF1 in polysome fractions was assessed by Western blot analysis. The blots are representative of 2 independent experiments. **e** Timing of the KD and MRZ treatment applied in panel (**f**). **f** Western blot analysis of RNF10, Ub$_1$-RPS3 and Ub-K48 24 h after KD of RPL7 (S301), RPL11 (S304) or RPL5 (S310) in comparison to control KD (S75) in HeLa cells, ± treatment with the proteasome inhibitor marizomib (MRZ, 8 h, 100 nM), Tubulin serves as loading control. The blot is representative of 2 independent experiments. **g** RNF10 protein levels were quantified from Western blots as in panel (**f**), shown are mean values ± SD (n = 2 biological replicates; p values determined by two-tailed, paired t test). Source data are provided as a Source Data file.

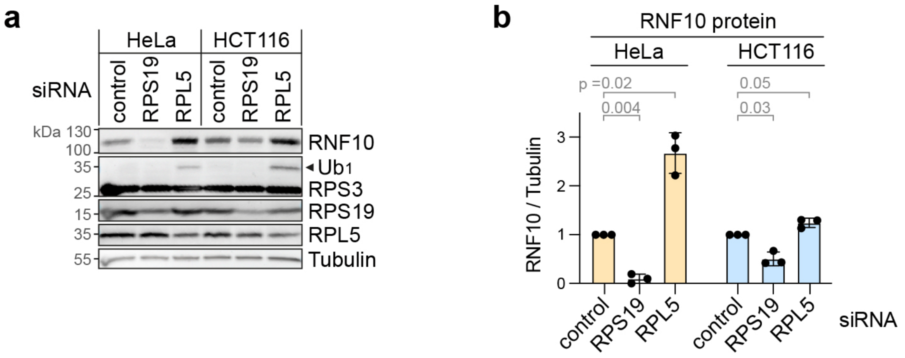

Supplementary Fig. 6 | RNF10 expression in HeLa and HCT116 cells after RPS and RPL KD.
a Western blot analysis of RNF10 and Ub_1-RPS3 in HeLa and HCT116 cells 24 h after KD of RPS19 (siRNA S309) or RPL5 (S310) in comparison to control KD (S75). The blot is representative of 3 independent experiments. **b** RNF10 protein levels were quantified from Western blots as in panel (**a**), shown are mean values ± SD (n = 3 biological replicates; p values determined by two-tailed, paired t test). Source data are provided as a Source Data file.

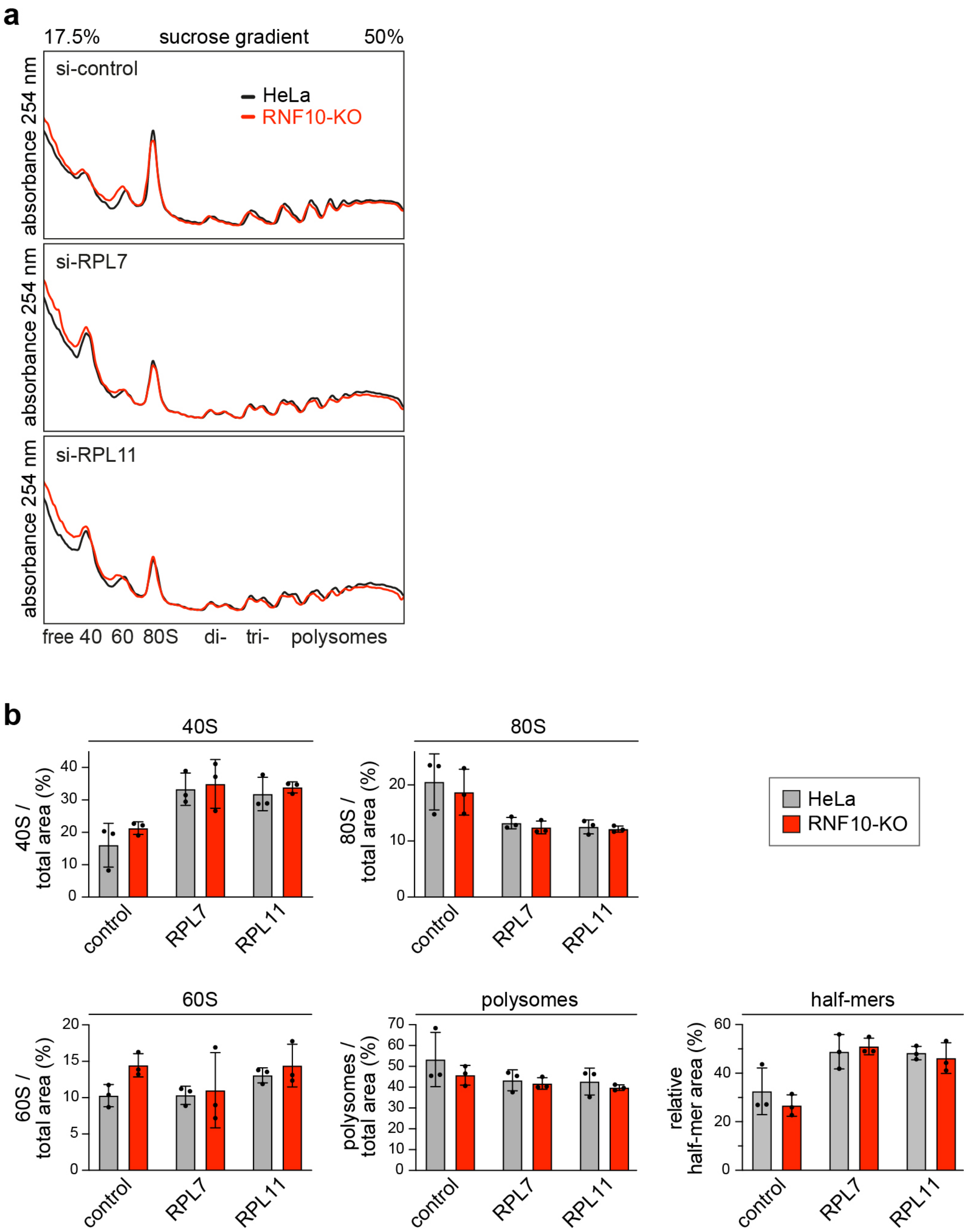

Supplementary Fig. 7 | Ribosomal half-mer formation upon RPL KD.
a Polysome profile analysis of HeLa cells and HeLa-RNF10-KO cells (clone 29-7) 12 h after KD of RPL7 (siRNA S301) or RPL11 (S304) in comparison to control KD (S75). **b** Polysome profiles as in panel (**a**) were quantified by calculating the area under the 40S, 60S, and 80S peaks as well as under the polysomal part of the profile, half-mers (2.5-mer / disome) were quantified as shown in (**Fig. 2d**) (mean ± SD; n = 3 biological replicates). Source data are provided as a Source Data file.
