# Supplementary material for: E3 ubiquitin ligase RNF10 promotes dissociation of stalled ribosomes and responds to ribosomal subunit imbalance
Source: Nat Commun. 2024 Nov 28;15:10350. doi: 10.1038/s41467-024-54411-x (PMC11604940; doi:10.1038/s41467-024-54411-x)
Supplement: Supplementary file 2 — Reporting Summary [file 41467_2024_54411_MOESM2_ESM.pdf]

nature portfolio

Corresponding author(s): Georg Stoecklin
NCOMMS-22-43495A

Last updated by author(s): Aug 13, 2024

Reporting Summary

Nature Portfolio wishes to improve the reproducibility of the work that we publish. This form provides structure for consistency and transparency in reporting. For further information on Nature Portfolio policies, see our Editorial Policies and the Editorial Policy Checklist.

Statistics

For all statistical analyses, confirm that the following items are present in the figure legend, table legend, main text, or Methods section.

n/a	Confirmed	
☐	☒	The exact sample size (*n*) for each experimental group/condition, given as a discrete number and unit of measurement
☐	☒	A statement on whether measurements were taken from distinct samples or whether the same sample was measured repeatedly
☐	☒	The statistical test(s) used AND whether they are one- or two-sided<br>*Only common tests should be described solely by name; describe more complex techniques in the Methods section.*
☒	☐	A description of all covariates tested
☒	☐	A description of any assumptions or corrections, such as tests of normality and adjustment for multiple comparisons
☐	☒	A full description of the statistical parameters including central tendency (e.g. means) or other basic estimates (e.g. regression coefficient) AND variation (e.g. standard deviation) or associated estimates of uncertainty (e.g. confidence intervals)
☐	☒	For null hypothesis testing, the test statistic (e.g. *F*, *t*, *r*) with confidence intervals, effect sizes, degrees of freedom and *P* value noted<br>*Give P values as exact values whenever suitable.*
☒	☐	For Bayesian analysis, information on the choice of priors and Markov chain Monte Carlo settings
☒	☐	For hierarchical and complex designs, identification of the appropriate level for tests and full reporting of outcomes
☒	☐	Estimates of effect sizes (e.g. Cohen's *d*, Pearson's *r*), indicating how they were calculated

Our web collection on statistics for biologists contains articles on many of the points above.

Software and code

Policy information about availability of computer code

Data collection	Collection of Western Blot images: Evolution-Capt V18 Software Vilber<br>Collection of qRT-PCR data: QuantStudio 5 Software (Thermo Fisher Scientific)<br>Collection of polysome profiles: PeakTrak vl.10 Software;<br>Collection of FACS data: BD FACS Diva software v9.0.1
Data analysis	Quantification of Western Blots: ImageJ vl.52<br>DNA and amino acid sequence alignments: Clustal Omega<br>FACS analysis: FlowJo software v7.6.5<br>Data visualization and analysis: GraphPad Prism v 8.4.l,Microsoft Excel 2019<br>Polysome profile alignment, normalization and quantification: QuAPProApp, https://doi.org/10.1101/2024.05.02.592260

For manuscripts utilizing custom algorithms or software that are central to the research but not yet described in published literature, software must be made available to editors and reviewers. We strongly encourage code deposition in a community repository (e.g. GitHub). See the Nature Portfolio guidelines for submitting code & software for further information.

Data

Policy information about availability of data

All manuscripts must include a data availability statement. This statement should provide the following information, where applicable:

- Accession codes, unique identifiers, or web links for publicly available datasets
- A description of any restrictions on data availability
- For clinical datasets or third party data, please ensure that the statement adheres to our policy

Source data of experiments shown in Figures 1a-d, 2a-g, 3a-c, 4a-e, 5a-g, 6a-b, 7a-c, 8a-c, in the Supplementary Figures 1a-i, 2a-g, 3, 4a-i, 5a-g, 6a-b, 7a-b, and in the Source Data File.

Research involving human participants, their data, or biological material

Policy information about studies with human participants or human data. See also policy information about sex, gender (identity/presentation), and sexual orientation and race, ethnicity and racism.

Reporting on sex and gender	*Use the terms sex (biological attribute) and gender (shaped by social and cultural circumstances) carefully in order to avoid confusing both terms. Indicate if findings apply to only one sex or gender; describe whether sex and gender were considered in study design; whether sex and/or gender was determined based on self-reporting or assigned and methods used. Provide in the source data disaggregated sex and gender data, where this information has been collected, and if consent has been obtained for sharing of individual-level data; provide overall numbers in this Reporting Summary. Please state if this information has not been collected. Report sex- and gender-based analyses where performed, justify reasons for lack of sex- and gender-based analysis.*
Reporting on race, ethnicity, or other socially relevant groupings	*Please specify the socially constructed or socially relevant categorization variable(s) used in your manuscript and explain why they were used. Please note that such variables should not be used as proxies for other socially constructed/relevant variables (for example, race or ethnicity should not be used as a proxy for socioeconomic status). Provide clear definitions of the relevant terms used, how they were provided (by the participants/respondents, the researchers, or third parties), and the method(s) used to classify people into the different categories (e.g. self-report, census or administrative data, social media data, etc.) Please provide details about how you controlled for confounding variables in your analyses.*
Population characteristics	*Describe the covariate-relevant population characteristics of the human research participants (e.g. age, genotypic information, past and current diagnosis and treatment categories). If you filled out the behavioural & social sciences study design questions and have nothing to add here, write "See above."*
Recruitment	*Describe how participants were recruited. Outline any potential self-selection bias or other biases that may be present and how these are likely to impact results.*
Ethics oversight	*Identify the organization(s) that approved the study protocol.*

Note that full information on the approval of the study protocol must also be provided in the manuscript.

Field-specific reporting

Please select the one below that is the best fit for your research. If you are not sure, read the appropriate sections before making your selection.

☒ Life sciences ☐ Behavioural & social sciences ☐ Ecological, evolutionary & environmental sciences

For a reference copy of the document with all sections, see nature.com/documents/nr-reporting-summary-flat.pdf

Life sciences study design

All studies must disclose on these points even when the disclosure is negative.

Sample size	No statistical methods were used to predetermine sample size. Essential experiments were performed at least in triplicates. Triplicate experimental design enables detection of outliers and is sufficient to determine statistical significance.
Data exclusions	No data has been excluded from the analyses.
Replication	All data are presented as mean ± SD if not indicated otherwise. Experiments were repeated independently, the number or repeats (n) and the type of statistical test is indicated in the figure legends.
Randomization	Randomization was irrelevant, because sample assignment was determined by the treatment of the biological material and is therefore an intrinsic property of the sample.
Blinding	Blinding was irrelevant, because the analysis of the data left no room for subconcious manipulation due to any preconceived expectations concerning its outcome.

Reporting for specific materials, systems and methods

We require information from authors about some types of materials, experimental systems and methods used in many studies. Here, indicate whether each material, system or method listed is relevant to your study. If you are not sure if a list item applies to your research, read the appropriate section before selecting a response.

Materials & experimental systems

n/a	Involved in the study
☐	☒ Antibodies
☐	☒ Eukaryotic cell lines
☒	☐ Palaeontology and archaeology
☒	☐ Animals and other organisms
☒	☐ Clinical data
☒	☐ Dual use research of concern
☒	☐ Plants

Methods

n/a	Involved in the study
☒	☐ ChIP-seq
☐	☒ Flow cytometry
☒	☐ MRI-based neuroimaging

Antibodies

Antibodies used

For Western Blotting: Rabbit anti-RPS6 (5G10; Cell Signaling, 2217), rabbit anti-RPS3 (Bethyl, A303-840A), rabbit anti-RPS19 (Bethyl, A304-002A), rabbit anti-RPS20 (Abcam, ab133776), rabbit anti-RNF10 (Proteintech, 16936-1-AP), rabbit anti-ZNF598 (Sigma, HPA041760), mouse anti-Tubulin (Abcam, ab6160), rabbit anti-RPL7 (Proteintech, 14583-1-AP), rabbit anti-RPL5 (Cell signaling, 14568), mouse anti-RPL11 (Thermo Fisher Scientific, 37-3000), mouse anti-p53 (DO-1, Santa Cruz), mouse anti-FLAG (M2, Sigma, F3165); rabbit anti-EDF1 (Abcam, ab174651), rabbit anti-phospho-histone H3 (Ser10) (Cell Signaling, 9701), rabbit anti-histone H3 (Abcam, ab1791), mouse anti-puromycin (Millipore, MABE343), and rabbit anti-ubiquitin-K48 (Abcam, ab140601).

Validation

Antibodies used in this study were previously used in the literature, validated by the manufacturer or validated in this manuscript.
anti- RPS6 (5G10; Cell Signaling, 2217) - Manufacturer's statement on specificity (WB): "S6 Ribosomal Protein (5G10) Rabbit Monoclonal Antibody detects endogenous levels of total S6 ribosomal protein independent of phosphorylation". This antibody was validated in https:// doi.org/10.1186/s13578-020-00422-2.
anti-RPS3 (Bethyl, A303-840A) - Manufacturer's statement on specificity (WB): "The epitope recognized by A303-840A maps to a region between residue 1 and 50 of human Ribosomal Protein S3 using the numbering given in entry NP_000996.2 (GeneID 6188)". This antibody was validated in this manuscript, in https://doi.org/10.1016/j.celrep.2021.109642 and in https://doi.org/10.1016/j.celrep.2021.109468.
anti-RPS19 (Bethyl, A304-002A) - Manufecturer's statement on specificity (WB): "The epitope recognized by A304-002A-T maps to a region between residue 95 to 145 of human Ribosomal Protein S19 using the numbering given in entry NP_001013.1 (GeneID 6223)". This antibody was validated in this manuscript.
anti-RPS20 (abcam, ab133776) - Manufecturer's statement on specificity (WB): "Predicted molecular weight at 13 kDa". This antibody was validated in this manuscript.
anti-RNF10 (Proteintech, 16936-1-AP): This antibody was validated by knockdown and knockout of RNFl0 in this manuscript and in https://doi.org/10.1016/j.celrep.2021.109468.
anti-ZNF598 (Sigma, HPA041760): This antibody was validated by knockdown of ZNF598 in this manuscript.
anti-Tubulin (abcam, ab6160): This antibody was validated in https://doi.org/10.15252/embr.202051851 and https://doi.org/10.1016/j.molcel.2016.08.030.
anti-RPL7 (Proteintech, 14583-1-AP) - This antibody was validated in https://doi.org/10.1016/j.bbrc.2021.10.059.
anti-RPL5 (Cell signaling, 14568) - Manufacturer's statement on specificity (WB): "RPL5 Antibody recognizes endogenous levels of total RPL5 protein".
anti-RPL11 (Thermo Fisher Scientific, 37-3000) - This antibody was validated in https://doi.org/10.4161/15384047.2014.955743.
anti-p53 (DO-1, Santa Cruz) - Manufacturer's statement on specificity (WB): "p53 (DO-1) is a mouse monoclonal antibody epitope mapping between amino acid residues 11-25 at the N-terminus of p53 of human origin". This antibody was validated in https://doi.org/10.1038/ s41586-019-0885-0 and in https://doi.org/10.1038/s41598-020-69499-6.
anti-FLAG (M2, Sigma, F3165) - This antibody was validated in this manuscript and in https://doi.org/10.1038/s41467-021-27471-6.
anti-EDF1 (Abcam, ab174651) - This antibody was validated in https://doi.org/10.7554/eLife.58828.
anti-phospho-histone H3 (Ser10) (Cell Signaling, 9701) - This antibody was validated by the manufacturer´s statement: "Detects a band approximately at 17 kDa", in https://doi.org/10.1128/MCB.23.5.1808-1816.2003, and in https://doi.org/10.1038/sj.onc.1210787.
anti-histone H3 (Abcam, ab1791) - This antibody was validated in https://doi.org/10.1038/s41388-021-01664-1 and in the manufacturer´s statement on WB specificity: "Detects a band of approximately 17 kDa".
anti-puromycin (Millipore, MABE343) - This antibody was validated in this manuscript and by the manufacturer: "The antibody was controlled by HEK293 cell lysates treated with Puromycin and Cyclohexamide, or with Puromycin only".
anti-ubiquitin-K48 (Abcam, ab140601) - This antibody was validated by the manufacturer´s statement "This antibody only recognizes polyubiquitin chains formed by Lys-48 (K48) residue linkage".

Eukaryotic cell lines

Policy information about cell lines and Sex and Gender in Research

Cell line source(s)

The following cells were a kind gift from:

Cell line source(s)	Hela and HEK293 cells - Paul Anderson, Harvard Medical School, Boston, Massachusetts, USA.<br>RPE1 cells - Ingrid Hoffmann, German Cancer Research Center Heidelberg, Germany.<br>HCT116 cells - Bert Vogelstein, Johns Hopkins Medical School, Baltimore, Maryland, USA.
Authentication	Hela, HEK293, RPE1, and HCT116 cells were authenticated via SNP profiling by Multiplexion GmbH at DKFZ.
Mycoplasma contamination	All cell lines were tested for mycoplasma contamination using the PCR Mycoplasma Test Kit (AppliChem).
Commonly misidentified lines<br>(See ICLAC register)	None.

Plants

Seed stocks	*Report on the source of all seed stocks or other plant material used. If applicable, state the seed stock centre and catalogue number. If plant specimens were collected from the field, describe the collection location, date and sampling procedures.*
Novel plant genotypes	*Describe the methods by which all novel plant genotypes were produced. This includes those generated by transgenic approaches, gene editing, chemical/radiation-based mutagenesis and hybridization. For transgenic lines, describe the transformation method, the number of independent lines analyzed and the generation upon which experiments were performed. For gene-edited lines, describe the editor used, the endogenous sequence targeted for editing, the targeting guide RNA sequence (if applicable) and how the editor was applied.*
Authentication	*Describe any authentication procedures for each seed stock used or novel genotype generated. Describe any experiments used to assess the effect of a mutation and, where applicable, how potential secondary effects (e.g. second site T-DNA insertions, mosiacism, off-target gene editing) were examined.*

Flow Cytometry

Plots

Confirm that:

☒ The axis labels state the marker and fluorochrome used (e.g. CD4-FITC).

☒ The axis scales are clearly visible. Include numbers along axes only for bottom left plot of group (a 'group' is an analysis of identical markers).

☒ All plots are contour plots with outliers or pseudocolor plots.

☒ A numerical value for number of cells or percentage (with statistics) is provided.

Methodology

Sample preparation	Cells were trypsinized, resuspended in 1% [v/v] FBS/1xPBS, washed with 1xPBS, resuspended in FACS buffer (10 mM HEPES, pH 7.2; 0.5 mM EDTA, 2% BSA) and 1.25 nM Sytox Red Dead Cell Stain (Invitrogen, S34859). Cells were then transferred to round-bottom polystyrene test tubes through cell strainer snap caps.
Instrument	FACSAria IIIu BD Fusion Cell Sorter
Software	Collection of FACS data: BD FACS Diva software v9.0.1<br>FACS analysis: FlowJo software v7.6.5
Cell population abundance	Information about the abundance of the relevant cell populations within post-sort fractions as well as providing details on the purity of the samples does not apply to our experiments since we only measured the cells for GFP and mCherry fluorescence and did not use those cells for further experiments.
Gating strategy	1) FSC-A/SSC-A gating of single cell population (P1), excluding debris and doublets.<br>2) Sytox Red APC-A/SSC-A gating into viable and dead cells.<br>3) GFP-A/mCherry PE-Texas Red-A gating of viable cells into GFP positive and mCherry positive cells in quadrant Q2, excluding autofluorescent GFP negative and mCherry negative cells.<br><br>The detailed gating strategy is shown in Suppl. Fig. 2e and in the Source Data File.

☒ Tick this box to confirm that a figure exemplifying the gating strategy is provided in the Supplementary Information.
